# American Dental Association

**Published:** 1892-03

**Authors:** 


### Reports of Society Meetings.

### AMERICAN DENTAL ASSOCIATION.

*August 6, 1891.—Third Day.—Morning Session (Continued).*

### PROSTHETIC DENTISTRY, CHEMISTRY, AND METALLURGY.

SECTION I.

DR. STUBBLEFIELD read a paper on the effect of hydrogen peroxide, of which the following is an abstract:

### HYDROGEN PEROXIDE.

Last year, as you will remember, Professor Peirce presented a tooth which had been subjected to the action of hydrogen peroxide, which showed most wonderful results. In the discussion which followed, the writer, knowing that hydrogen peroxide was made by the action of peroxide of barium upon hydrochloric acid, guessed that the component part which caused the irritation of the tooth had been an excess of that acid. During the winter he had made more careful examinations. He had obtained from New York, Brooklyn, and St. Louis fresh, unbroken packages, when possible, but certainly fair samples of the three best makes of hydrogen peroxide. It was an easy matter to detect in all three the presence of not only hydrochloric acid, but also sulphuric acid. Not satisfied with himself, he went to Dr. Dudley, professor of chemistry in the Vanderbilt University, and submitted to him three samples of the agent, as above. His examination revealed a large percentage of HCL, and by another test a large quantity of $H_2SO_4$ was found ; he also found a whitish residue that was charred by heating strongly in the platinum cup. It may be thought these discoveries amount to little, but they rise in importance when considered in connection with the statement of each manufacturer that his product is absolutely pure $H_2O_2$. One claims, in his circular accompanying original packages, that he is the sole manufacturer of chemically pure $H_2O_2$, to be used medicinally, yet it can be shown from two original packages, that both HCL and $H_2SO_4$ were present. He claims, also, that ozone is the active agent of his $H_2O_2$.

It is not stated that his is not a preparation of $H_2O_2$. There are other things in it, and these other things change its nature necessarily. If the profession be restricted to the use of such defective products over signatures, we wonder that results are so strangely favorable.

Now, as to the phenomena observed by Professor Peirce: Any one of those three had sufficient HCL to dissolve a tooth after sufficient exposure to its action ; but the calcium chloride formed by it, being extremely soluble in water, would not appear as a white mass. On the other hand, any one of those three had sufficient $H_2SO_2$ present to dissolve the tooth after sufficient maceration, and the calcium sulphate formed, being insoluble in water, would precipitate in a white mass at the bottom of the tube.

Further still, as the acid grew weaker, the sulphate would coat the tooth and retard, or even stop, the action, thus accounting for what he observed.

As to the other inquiry of his, the tooth was attacked by the acids present, though not so irregularly as he had expected. This was settled at the time by several good observers.

In conclusion, it is desired to present the results of some reflection and experiments on the subject by your essayist.

The distrust awakened by such flagrant departures from the statements of the manufacturers naturally aroused in his mind the question whether we dentists ever had used any actually pure hydrogen peroxide. Has any one here ever used any but the chemical products of our best manufacturers? If not, then it is claimed we have always used a solution of $H_2O_2$ with acids present. This is about the only claim urged by this contribution, and it is presented to this body for consideration merely. The experiments in this connection have not been as thoroughly and extensively pursued as desired, but they may be properly presented as a report of progress in the matter.

The $H_2O_2$ as obtained from the original package or fresh supply was used in pulpless teeth, where pus was observed, and the effervescence was noted. On the very same occasion it was used in the canal of a sound tooth which had been cleaned out for the purpose, and the same evolution of gas was again observed. The point that was apparently made by this was that the agent would cause this phenomenon when used with pus or without it, when inside of a tooth. This experiment was repeated several times with substantially the same result.

Then the attempt was made to obtain this evolution of gas when the $H_2O_2$ was put in contact with pus outside and away from a tooth. Pus was obtained from gum-margins and pulp-chambers and subjected to this same specimen of $H_2O_2$. As far as could be observed, there was no evolution of gas. This was repeated, also with the same results.

It may be stated, parenthetically, that the tooth in the mouth is heated to about ninety-nine degrees, which would be sufficiently high to cause the rapid disengagement, comparatively, of the loosely combined oxygen, and the difference of temperature outside the mouth might account in some respects for the tardiness of action observed when the tooth was in the hand; but this does not account for the failure to obtain the same evolution of gas away from pus that may be seen when the pus is in a tooth.

The next step was to free the $H_2O_2$ from all acids, that is, HCL and $H_2SO_4$. When test failed to indicate their presence, it was carefully preserved for experiment. In each case this observer failed to see any evolution of gas whatever in the canal, or out of it, in contact with pus, or away from it, in the mouth or in the tooth out of it. This was repeated with the same results. His failure to obtain any pus at the City Hospital, from a neighboring surgeon, was a disappointment, for he desired to compare results from action on pus pure and simple, with action on the mixture of pus and saliva found in the mouth. This he reserved for a better opportunity that never came.

The last experiment was with the HCL itself, used almost full strength. It produced almost the identical phenomena as those by this $H_2O_2$ in the first place,—evolution of gas and all.

DISCUSSION.

*Professor Peirce.*—I would like to say only one word in extenuation of the subject. The tooth I placed in the solution I think at the time was a tooth largely affected by excementosis, and the surprise to me was that this abnormal growth was rapidly acted upon until it was all removed from the root of the tooth, without any perceptible action upon the crown or the enamel of the tooth. When disintegration had gone to the extent of removing the cementum of the tooth, and leaving only the dentine and enamel exposed, the action was very slow, and hardly perceptible. The tooth I presented here to the Association had only the enamel and the dentine exposed, the cementum having all been removed, and my surprise was that it had not acted upon the tooth while acting so rapidly upon the cementum.

*Dr. Abbott.*—I am much pleased with this paper, but still it is sad to feel that for so many years we have been using peroxide of hydrogen, and been deluded into the idea that it was a test for the presence of pus. It seems from the paper that it is not a test at all, and I have no doubt that many other substances we purchase would produce the same result in the pulp-canal of a tooth. Outside of the tooth they act the same,—no disturbance, no indication that it has any effect upon the pus. I say, while it is a very excellent matter to investigate and to learn, and particularly to observe such things, still it is to be regretted that we have been deluded so long.

*Dr. Volck.*—As peroxide of hydrogen is used, I think we will find it is hardly a chemical preparation. It is merely a mixture of another compound, and I have made a few experiments by allowing a bottle of it to stand open for a couple of hours, and I did not find anything in it but water. I doubt very much whether there is much value in it.

*Dr. Freidrichs.*—I am sorry to acknowledge that I was cognizant that the peroxide of hydrogen that I was using was not pure,—absolutely pure. It takes a very little heat to break it up, and, as mentioned by previous speakers, when injected into the tooth at 90° heat it effervesces. So when you drink a glass of soda-water you see the carbonic acid flying off. I think we have a good use for it, however. In the first place, the combination of hydrochloric acid and sulphuric acid is in that proportion that they have a tendency to stimulate and heal. When you have an abscess where it is difficult to discharge the pus from it, what better agent could you desire? You inject it in there, and the heat disorganizes it, and consequently it forces out the pus and the fluids that are contained in the sac, the same as powder drives out the shot from a gun.

*Dr. Truman.*—While I am pleased with the results of Dr. Stubblefield's work, I shall continue to use it the same as I have done heretofore, for the reason stated by Dr. Freidrichs. I have found it one of the most valuable agents. I think it would be a great mistake to abandon its use. In cases of alveolar abscess that have failed to heal under other treatment, I have found that it gives us the best results; and I know of no agent as yet that can act in necrosis of the jaw with the same good effect as this one.

This agent has been before the profession for several years, and, while it has been severely judged to-day, let us remember its good work in the past, and not place it, as many do, in an atmosphere to become deteriorated, above 60°. It then becomes water. It must be kept cold. I do not fear the agents that are mixed with it, because they are all good in their places. I want it as a germicide, and I believe we have no better agent than hydrogen peroxide for that purpose.

*Dr. Ingersoll.*—I have long felt what has just been stated, that the possibility is that it is not a chemical compound. Of course, oxygen is retained by force. Its liberation is caused by heat, and not by chemical action upon the pus. I have tried it many times, but I have never had the confidence in peroxide of hydrogen that some have, for the reason that it lacks one element that is almost essential in all drugs, and that is a stimulating property. It should therefore be used in connection with some stimulating agent, because in almost every disease we need such a property. I have observed another thing in regard to it,—it acts badly in some mouths where there is an antral disease. If the opening is closed, and the tube is inserted to give artificial drainage, a cheese-like sediment forms by the action upon the pus, and it acts as an irritant and causes great pain. I question very much its use in the pockets of pyorrhœa alveolaris, from the fact that it is absolutely essential that it come in contact with the pus at every point. The difficulty is in compelling it to get in contact. It should be used on cotton, thoroughly saturated with it. I think we are inclined to accept every medicine offered, and reject the old, which have been successfully used.

*Dr. Horton.*—I want to say a word with reference to what Dr. Ingersoll stated about the cheese-like sediment that forms. I take some warm water and chloride of sodium, and carefully syringe out the pockets where this pus is supposed to be prior to using the peroxide, and I obtain very good results.

*Dr. Marshall.*—Dr. Ingersoll opened a question that I have had some experience in. He spoke of the action of the peroxide. I have had the same experience with it that he has had, and have abandoned its use on that account.

I have also abandoned its use in cases of alveolar abscess. I will give one illustration. I attempted to treat a case of a lower molar, and tried to open into the pulp chamber. The gentleman who had made the operation had been so unfortunate as to pass the drill through the tooth, while attempting to open it. He put in some arsenic, and of course the arsenic went through, and we had necrosis of the septum between the roots. There had been discharges from the outside of the face. I injected the peroxide of hydrogen, and it did not seem to do much good. My patient was nearly frantic. I took a lancet and cut down both sides of the opening to give exit to the gas, but had a bad case of cellulitis that took several weeks to heal. I said that is the last time I will use peroxide of hydrogen, and it has had no place in my office since.

*Dr. Stubblefield.*—Dr. Ingersoll speaks of the lack of stimulating action. I am of the opinion that those acids supply sufficient. Still, his experience may not agree with mine. I have found that where there were pus-pockets, if I could get down with a little sharpened stick, carrying the acid far down, it was followed by a healthy growth and resumption of a healthy condition.

Regarding the excessive pain after the injection into the antrum, I believe it is the sulphuric acid brought in contact with water. You know in handling sulphuric acid and diluting it we have to add the acid to the water; if you do it the other way you will have an explosion. If it is too closely confined, we have that change and effervescence which is probably a coagulation, and then this elevation of temperature will be brought about.

### BANDING ROOTS.

*Dr. Parmly Brown.*—The question of banding all kinds of crown- and bridge-work, is still an open one. I can give you a few illustrations of the condition that it is in to-day. When an eminent practitioner in this country comes to New York and brings an entire upper and lower set of natural teeth, a few days before extracted from the mouth of a lady in his city, which had been crowned and bridged at an expense of six hundred dollars, and asks us not to get too near it for fear we might observe something that may be serious,—when he said, "I extracted these teeth to save her life, after consultations with eminent physicians," it means that this is still an open question, and very wide open. Last year a lady came to my office and said she had bands driven up around her teeth until she got out of the chair and said, as Charlotte Cushman or any of our best tragedians would say, "I will be a raving maniac if you put another band on my teeth."

Another lady came to me and said, "I was sitting in the office of a large establishment, and an army officer came in, as if war had been declared. He said, 'Take out those bands from my mouth, or I'll blow out your brains.'" Now what does all this mean?

I have been an advocate of never banding if you can help it. When a band can be devised that will not touch nor injure that most delicate of all tissues in the system, the membrane around the necks of the teeth, then you can do more than you do now. In the present state of the art, there should be, if possible, never a gold band. If you have a gold band, you cannot bake it if occasion requires. It should always be a platinum band, because that will always stand the fire. It will do, in nine cases out of ten, where a gold band will not. I have put on a No. 11 band, and shall try to bake it. I have one of those platinum bands, which I do not say is new.

I take a piece of platinum from thirty to thirty-six, very thin, always as thin as possible. Wrap it around and lap the edge over. I find there are no two ends of roots alike. That is why this system is good for capping roots. Put a piece of thirty to sixty pure gold between the caps; add a little flux to it and solder it. Fit it in gently. Get the length to suit. Slip it right down like this. Put your electric plugger on (if you have one that is alive), or any kind of a plugger, and work it around there. When you get the case bent over in that way you have a perfect adaptation. This case, which I will pass around, is one we made day before yesterday, and you will notice that the band is not fastened on. In extreme cases, where there are reasons that you do not want to solder or bake, you can use this, and you have the ordinary crown fastened on with the addition of this band, which is a separate arrangement. It protects the root somewhat. You can follow these bands in an undercut. In the case of the lady whose life was saved, the sulphur end of a parlor match would break up all the gold bands in her teeth. If you have one serious undercut, that platinum will go right under there, if you use a very fine point. You cannot put it up with two such undercuts opposite each other. In most of my cases I bake the porcelain on and cover up this band so that there is room for just a little film of porcelain. I have a lady here who has one of those on. Why did I band it? Because she had a bicuspid on the other side that was split. It is proper to cut off teeth for ladies where unsightly gold fillings show, if you can do it this way, so that there is no danger of splitting the root. You are justified in excising teeth that five years ago it would have been considered a serious case of malpractice to cut off. I look around here and I see four or five eminent dentists who were spoken of as being at my office. A lady came to me and said, "I want that tooth crowned." She mentioned the names of several eminent dentists whom, she said, would not crown it for her. They would not pull out a set of teeth, as our friend did, to save a lady's life. She came to me, told me what her name was, and said, "Now, Dr. Brown, you know what handsome teeth my sister has. Well, for a long time this tooth has been a horrible nightmare to me."

*Dr. Ames.*—I would like to offer a method of making a very close joint between gold and porcelain in crown- and bridge-work, by means of lapping thin, pure gold upon the surface of the porcelain, making an accurate joint, as you will see. The specimens were presented by Dr. J. H. Hollingsworth, of Kansas City, Missouri.

*Dr. Carroll.*—I desire to be very good-natured towards my friend, Dr. Brown, in discussing that crown, or his method of making it. The crown itself is all right, but the point I rise to speak upon is the cutting off of that lateral incisor to avoid an unsightly gold filling. I suppose it is to be a living lateral, and hence a very valuable tooth in its place. The root he spoke of in his remarks, or the method of it, is very correct, and he spoke of a justification of cutting off a lateral for the purpose of getting rid of an unsightly tooth. I have my method here, which I propose to pass around, the same lateral which he cut off, and I think it is pertinent in reply to his remarks. The sample which I shall exhibit is that of banding and covering a tooth or a root, and it shows a different method. I would not cut that lateral off in order to have a root to band, but I would do what is here done. Here is a case of a lady of New York, who thought we had no dentists in that city sufficiently capable of doing a fine piece of work, and went to Paris, where she consulted a very eminent practitioner, whom, I believe, we all know. Here is a sample of the case. The two front teeth were filled with gold on the anterior proximal corners; she would not allow him to contour on account of the unsightliness of the gold. The root was to be banded and a tooth inserted. The distinguished operator, formerly an American dentist, inserted a little piece of V-shaped porcelain to supply the anterior corners of these two front teeth. You will see what the result would be, that every motion of that lateral would necessarily make a motion of the little V-shaped porcelain piece that went to make up the two corners, and hence was unsightly. The case fell into my hands because of an abscess of the root. I removed the piece of porcelain and mounted a simple device there over those two teeth. Instead of cutting it off I telescoped over it with a porcelain front, backing it up with a little wax on the posterior part, and cast it in aluminum.

This will illustrate the amount of the cutting I did, which was very slight, and here is the form, all completed, which may not be new. It is original with me, and I am now doing a great deal of this reparation. I would also like to say that you will notice that the faces of this little bridge are defaced. I can explain that. I selected the shade of porcelain I wanted; it was very good. The tooth is a Weston crown. It was exactly the color, but when I put it in place she said it looked too shiny. I said I could take off the polish, but not put it back again. She told me to go ahead and take the shine off. I did so, and attempted to polish the others, and you see the result. I then got another manufacturer's tooth, which I crowned and polished over beautifully, so that this will represent the case as put into her mouth. I then cemented it in place, and it looked perfectly natural; and it is a perfect restoration without destroying the pulps of two valuable teeth.

### Section II.

### DENTAL EDUCATION, LITERATURE, AND NOMENCLATURE.

Report read by Dr. Ottofy, of which the following is an abstract:

Two members of the section have died during the past year.

The principal work of the section consisted in increasing interest in the local societies, that the reports of the committees may be made for the benefit of the entire profession.

A list of the dental societies and their members was prepared. It shows that there are at least one hundred and three dental societies scattered over the United States. There were represented in the society last year twenty-two, located in fifteen States and the District of Columbia; in other words, eighty-two societies and twenty-nine States were totally unrepresented.

A circular was issued by the Chairman of the Executive Committee, having for its object an awakening of the interest in local societies, and increased representation of them in this Association; also to secure a report of the different societies. It will be several years, however, before the plan will be perfectly carried out.

It is the intention of the committee to request each local society to prepare a list of its proceedings for the benefit of the Association, and to ask those societies which are not now represented by delegates to send representatives to the Association; also to publish a correct list of the dental societies and their members, now numbering about four thousand.

The Dental Association of the United States has established a course of study, which embraces a course of three years' reading of strictly dental works. On the completion of this the candidate is expected to pass the examination, and, if the reading has been thorough, he is then expected to take a practitioner's course in some dental college, upon the completion of which he is entitled to full membership in the Post-Graduate College. If he then pass a successful examination, a certificate to that effect will be issued to him.

The aim of that Association is to elevate and instruct the practitioner whose early education has not been as full as he might desire.

The subject of nomenclature has been referred to a committee of the dental meeting of 1893, to be composed of a council of dentists from all countries. We will not expect a report on this until the subject has been treated by a competent committee of dentists from all over the world.

The section reports one paper from Dr. Koch, of Chicago, entitled "State Boards, the People's Officers, and the Profession," read by Dr. Peirce, of which the following is an abstract:

### STATE BOARDS, THE PEOPLE'S OFFICERS, AND THE PROFESSION.

If we could only submit to the conditions that concern us, we would realize in the near future the desires that seem so far away. While we are citizens of the United States, we are also citizens of the several States in which we reside. By the laws of the Union, the right to make their own by-laws and regulations for the protection of their citizens has been reserved to the several States. They have also made laws that are meant for the protection of dentists, although they are not alone for the benefit of the dentists. They declare that these shall be competent to practise their profession.

It has been said that the status of the profession is established by the brightest light within it. People judge a calling by the men they know belonging to it, and are apt to give to it such esteem as is impressed upon their minds. If, therefore, we live so as to respect it, and have a desire that the laity should equally respect it, we must labor in a way that will cause them to honor and respect us. They do not care that a dentist is licensed by a national board. They demand that all dentists who shall hereafter offer their services to them shall commence with such a degree of education that they may intrust their teeth to the care of one who is fitted to act as a dentist.

The question would not be, "Has he the necessary funds to pay his tuition?" or, "Is he smart enough to make money?" No; the question is, "Has he the necessary qualifications of mind, body, and soul to serve the people faithfully in this particular sphere?"

If this rule be adhered to it would be a good thing. A diploma should not only be a *primâ facie*, but an absolute, evidence of the owner's capacities.

The State authorities, with two exceptions, exercise no supervision over the purely business enterprise of the dentists. They do not appoint or examine the teachers of the colleges. They authorize them to do such teaching as they think fit. In some States the boards are compelled to issue the license to practise to those who hold diplomas from any dental college. In other States the work of the colleges is reviewed by examiners. This is believed by the people to be the only safe way.

Much can be accomplished under the condition of the various laws as they now exist. A large majority of our colleges are private ventures, organized under some loosely-drawn law. All honor to the men and institutions whose aim has been to educate and protect the dentists. The institution in which education is ostensible only, which is conducted for revenue only, should be suppressed. In some colleges the tuition may be had for a very small sum. "The butcher, the baker, and the candlestick-maker" can be chartered as a dental college, and can issue diplomas which in some of our States have standing, and cannot be questioned. In most of our States, boards have been established with varied powers. These boards are intended for the protection of the people against imposition by dental colleges. They are the only representatives of the people. They are the only ones who are responsible for the enforcement of the law and the improvement of the dental service. It has been said that men who have never been in dental colleges themselves have been appointed on State Boards because of their skill in manipulation. This may be true. These boards are created to examine, not to teach. They are intended to enforce the laws; they require strong good sense, a knowledge of the laws, and a courage that shall give them power to report.

The manner of selection is capable of being objected to in the same way. Personally, the writer does not now recall an instance in which the appointee on the State Boards was not well and favorably known in his State as a good and conscientious practitioner. Is it unreasonable to believe that men who have had the affection and friendship of their colleagues will do aught that is not in the direction of elevation? Is it not reasonable to credit men who have nothing to gain, except by a faithful discharge of duty, with more circumspection, who must more or less be guided by questions of fact, and whose interest is largely of necessity concentrated within the walls of their own institution? It is far from the writer's intention to speak of teachers who have honestly and earnestly labored to put their colleges on a high standing. They should be separated from this class of pretenders.

It should be borne in mind that dental colleges have increased to a great extent since the people have begun to think of this matter. We have now nearly as many colleges in the land as we had graduates years ago. Is it proper that the people should demand a standard put on the manufacture of dental goods, too? There are people at all times who oppose law because it is law. Boards everywhere will tell you that the dental community has representatives of this class. The laws of the different States vary much on this subject, and many of them think it is impossible to do anything in the matter until it is controlled by a National Board.

A National Board is of course out of the question. There are matters of greater public concern than this which need legislation. It is common sense to accomplish the best with the opportunity you have to use the tools at your command, and to secure the best possible result with them. We should all, then, work in one direction. The future children of this family must be educated on a higher plane than their sires.

I urge upon you that you sustain both the law and its chosen executives to your utmost ability. I assure you that they occupy the position for the time being, and they can accomplish more with your aid and support than they can without it. If the position that has frequently been taken, that members of the board are many times themselves unfit to act as examiners, be true, I wish to say, lend them your aid, to construct better material for future years! I am quite sure that many of you have never had your thoughts directed to the fact that you discharge the obligations of your practice simply by the license of the people. The people, in the early history of the country did not see fit to assert their rights. The people have a right to demand that the physicians, the dentists, the lawyers, the teachers they employ, shall have the necessary qualifications to discharge their duty, and in many States this right is asserted.

If the people should not recognize any diploma whatsoever in dentistry, and subject every member to an examination, they would follow out the practice that is followed in some countries. Indifferent institutions and indifferent teachers would no longer be of any use. The degree of the Faculty ought to have more potency than the degree of the Board. The public is not satisfied with the evidence of a diploma, but requires a further examination by the Board, outside of the Faculty.

Nearly all our schools are private enterprises. The diploma and the tuition are both given for a consideration, and it is not unreasonable if the people, having the right so to do, through their officers, verify the qualifications of the dentists as certified on their diplomas.

The writer desires to express the wish that dentists generally would take a greater interest in the duties and trials that come to members of State Boards in the discharge of their duties; they would aid their colleagues who for the time being are in office.

If we would all do this, our profession would be much more respected, and the people would be better served, and when we finally lay down the forceps, the plugger, and the mallet, we would feel satisfied that we have used our instruments in the cause of general good.

(To be continued.)

